# Supplementary material for: The autophagy initiator ULK1 sensitizes AMPK to allosteric drugs
Source: Nat Commun. 2017 Sep 18;8:571. doi: 10.1038/s41467-017-00628-y (PMC5603566; doi:10.1038/s41467-017-00628-y)
Supplement: Supplementary file 1 — Supplementary Information [file 41467_2017_628_MOESM1_ESM.pdf]

Description of Supplementary Files

File name: Supplementary Information
Description: Supplementary figures and supplementary tables.

File name: Peer review file

Supplementary Fig 1

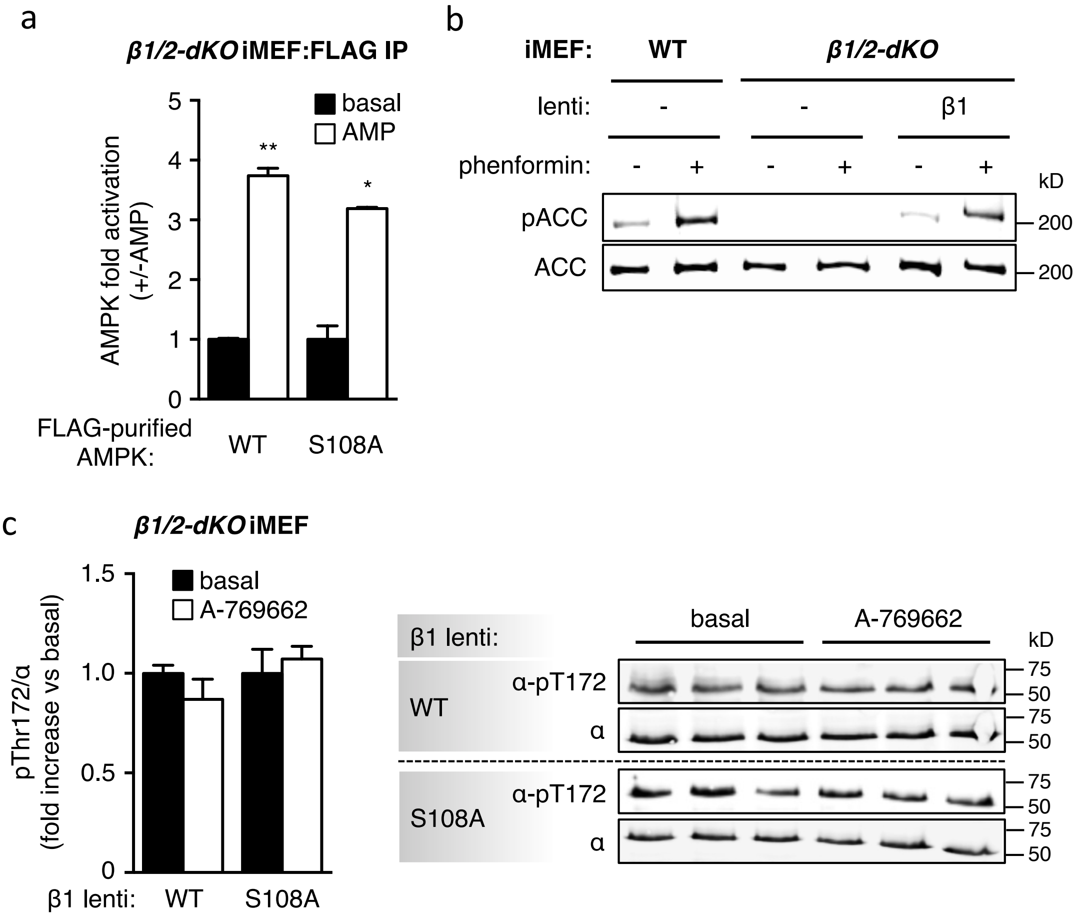

Supplementary Figure 1. Lentiviral transduction of AMPK β1 in β1/2 double knockout (*β1/2-dKO*) immortalized MEFs (iMEFs). (**a**) Activity of AMPK, purified from *β1/2-dKO* iMEFs expressing FLAG-fusions of either β1 WT or S108A mutant, in the presence of 200 μM AMP. n = 3. Error bars, mean fold AMPK activation relative to basal ± s.e.m. Statistical analysis was performed using unpaired two-tailed Student's t test. *P<0.05 and **P<0.01 indicate significant increase in AMPK activation compared to basal. (**b**) Immunoblot for pACC in lysates from WT or *β1/2-dKO* iMEFS, or *β1/2-dKO* iMEFs expressing β1 FLAG-fusion, stimulated with 1 mM phenformin for 45 min. n = 3, representative immunoblots shown. (**c**) Immunoblots for α-pThr172 in lysates from *β1/2-dKO* MEFs, expressing β1 WT or

S108A mutant, stimulated with 20 μM A-769662 for 90 min. $n = 3$. Error bars, mean fold increase in α-pThr172 increase relative to basal ± s.e.m.

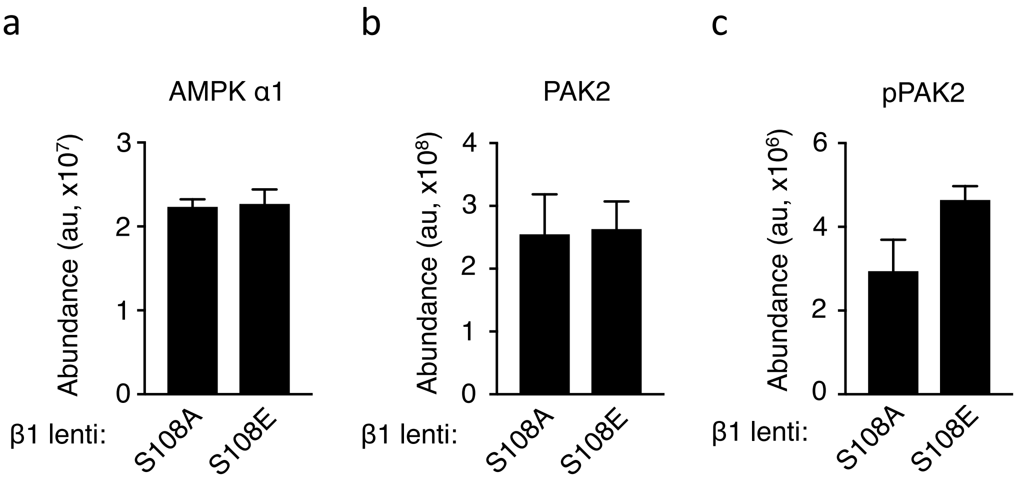

Supplementary Figure 2. Quantitative proteomic and phosphoproteomic analysis. Graphs show raw data peptide abundance (area under the curve) extracted from *β1/2-dKO* MEFs, transduced with either β1 mutants S108A or S108E. (**a**) AMPK α1 (comparative peptides: ^{467}SIDDEITEAK^{476} and ^{477}SGTATPQR^{484}) (n = 3), (**b**) PAK2 (13 comparative peptides across protein) (n = 3) and (**c**) pPAK2 (^{139}YLpSFTPPEKDGFPpSGTPALNTK^{160}) (n = 2).

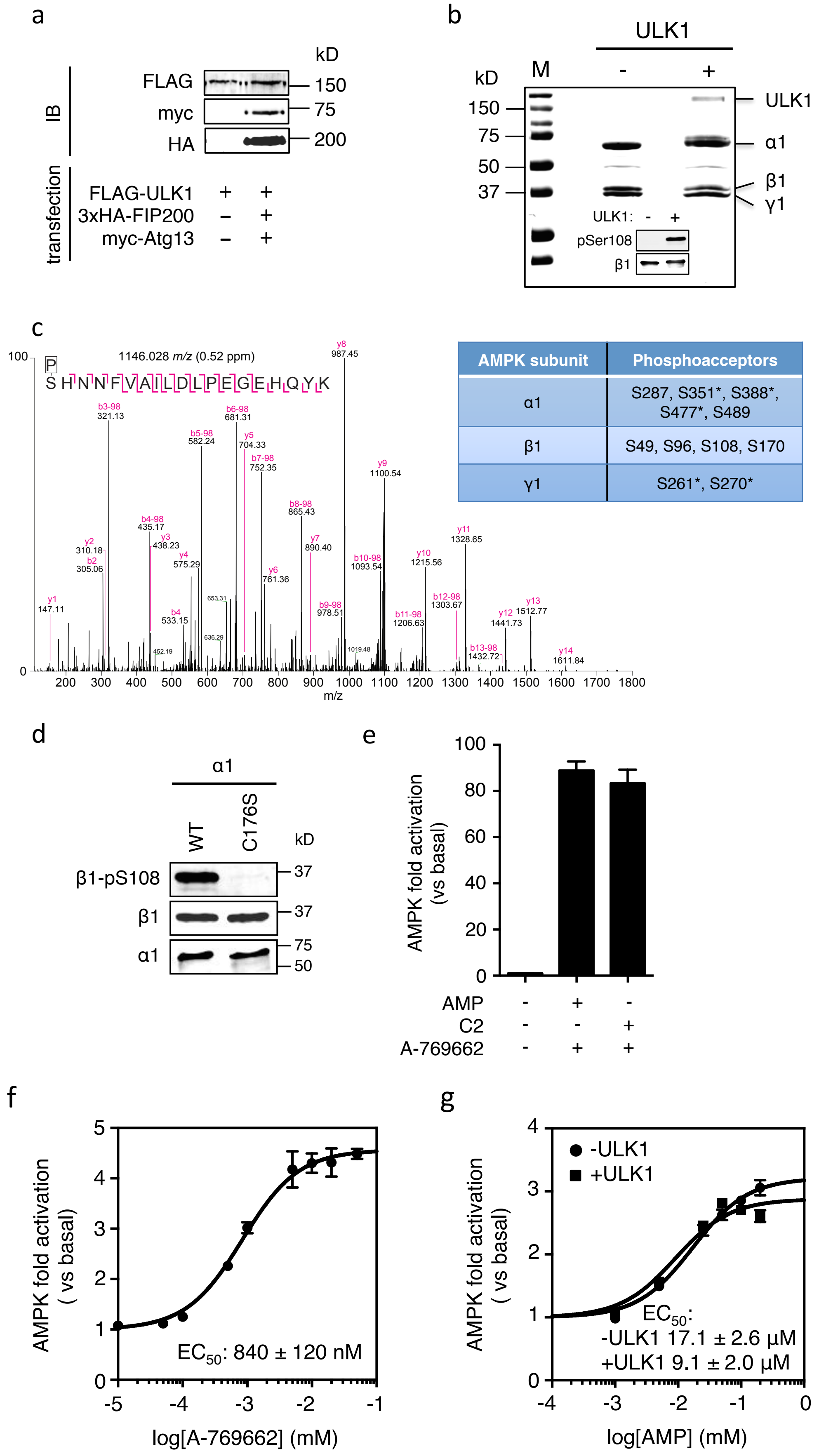

AMPK subunit	Phosphoacceptors
α1	S287, S351*, S388*, S477*, S489
β1	S49, S96, S108, S170
γ1	S261*, S270*

Supplementary Figure 3. Analysis of ULK1-treated AMPK. (a) Immunoblots for FLAG-ULK1, myc-Atg13 and HA-FIP200, transiently expressed in HEK293T in combinations as indicated. Preparations were used to phosphorylate S108tide synthetic peptide and purified AMPK. **(b)** Coomassie stained SDS-PAGE gel and β1-pSer108 immunoblot (inset) of purified, kinase inactive (KI)-AMPK phosphorylated with ULK1 for 30 min. AMPK subunit gel slices were analysed for phosphopeptides by LC-MS/MS. M: molecular weight markers. (**c**) LC-MS/MS analysis of ULK-treated KI-α1β1γ1. The masses of the b and y ion series provide direct evidence for phosphate incorporation onto β1-Ser108. Table shows other AMPK phosphosites detected at high confidence. *denotes previously detected[24]. (**d**) Immunoblot for β1-pSer108 in bacterial-expressed α1(C176S)β1γ1. (**e**) Activity of α1(C176S)β1γ1 co-incubated with 20 μM A-769662 and either 100 μM AMP or 0.1 μM C2. *n* = 3. Error bars, mean fold AMPK activation relative to basal ± s.e.m. (**f**) Dose curve for A-769662 activation of CaMKK2-treated α1(C176S)β1γ1. *n* = 3. Error bars, mean fold AMPK activation relative to basal ± s.e.m. (**g**) Dose curve for AMP activation of CaMKK2-treated α1(C176S)β1γ1 phosphorylated with ULK1. *n* = 3. Error bars, mean fold AMPK activation relative to basal ± s.e.m.

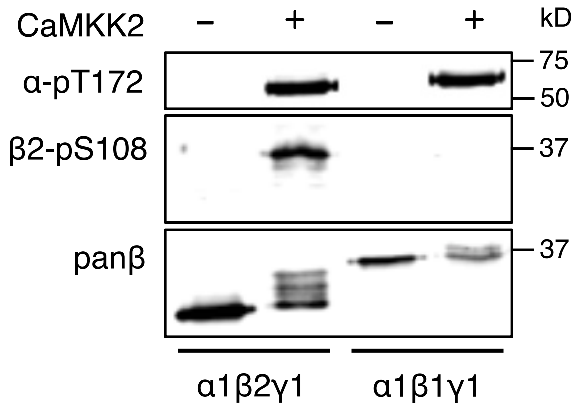

Supplementary Figure 4. Characterization of β2-pSer108 phosphospecific antibody. Immunoblots for α-pThr172 and β2-pSer108 in bacterial-expressed AMPK (α1β2γ1 or α1β1γ1), incubated with CaMKK2 to induce α-Thr172 phosphorylation and β-Ser108 autophosphorylation. The β2-subunit exhibits an extensive band shift upon autophosphorylation. β2-pSer108 phosphospecific antibody did not demonstrate cross reactivity against unphosphorylated β2 or autophosphorylated β1.

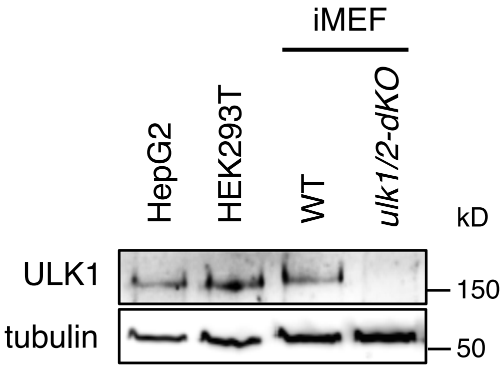

Supplementary Figure 5. ULK1 expression. Immunoblot for ULK1 in lysates from HepG2 and HEK293T cells, WT and *ulk1/2-dKO* iMEFs.

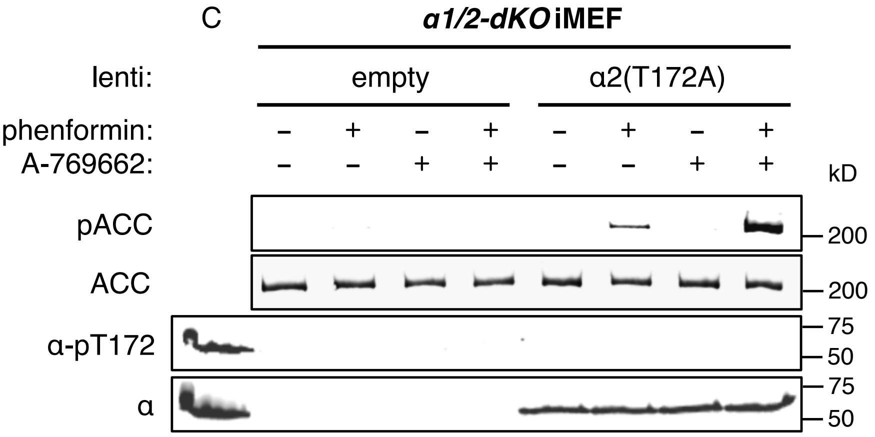

Supplementary Figure 6. AMPK is the exclusive upstream kinase for ACC-Ser79 in iMEFs in response to phenformin/A-769662. Immunoblots for pACC and α-pThr172 from *α1/2-dKO* iMEFs transduced with empty lentivirus or expressing α2 T172A mutant, stimulated with 2 mM phenformin and 100 μM A-769662 for 1 h. n = 3, representative immunoblots shown. C: CaMKK2-treated α1β1γ1 standard.

Fig1a

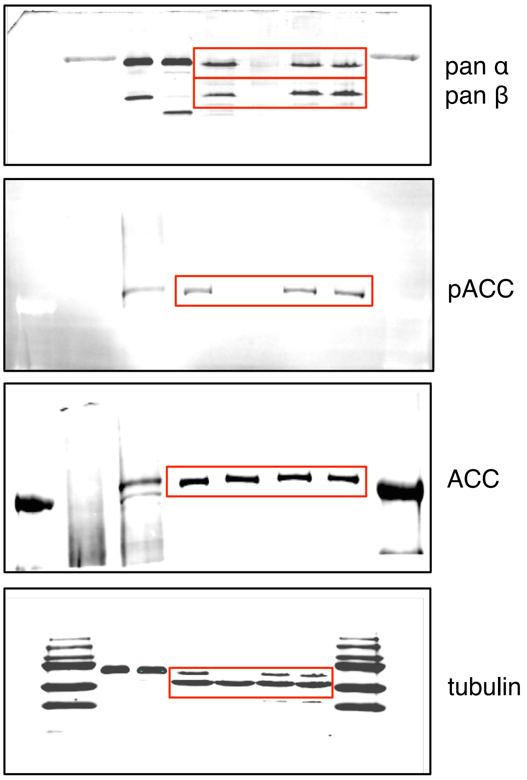

Fig1b

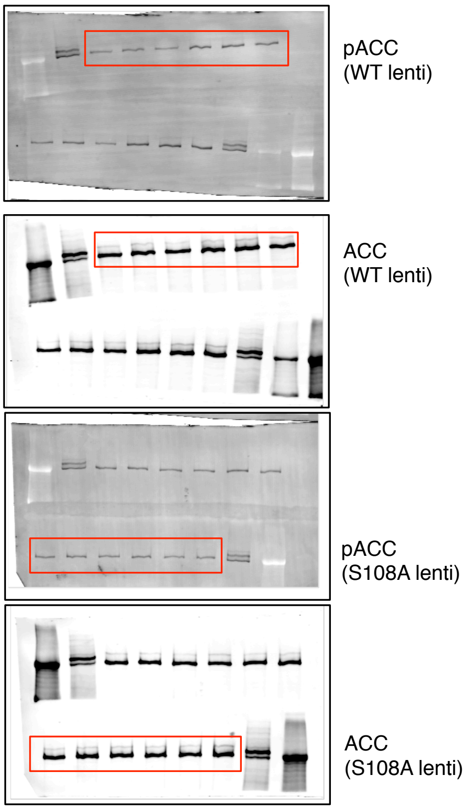

Fig2a

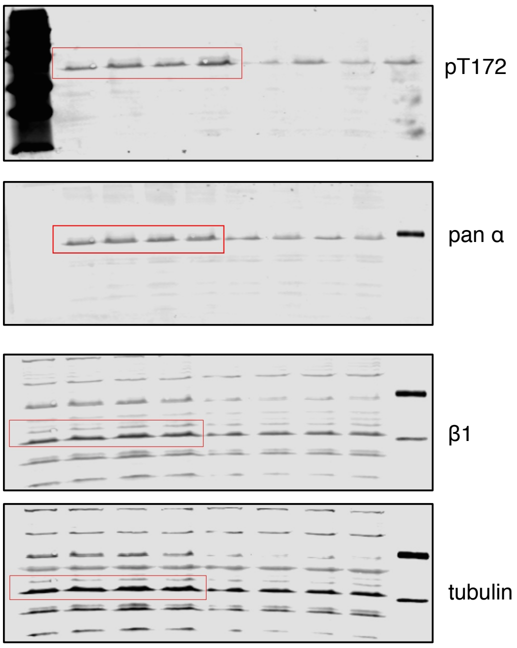

Fig2d

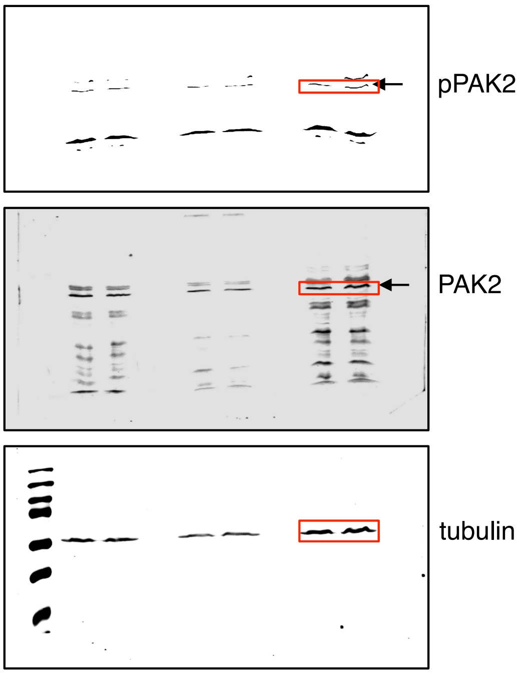

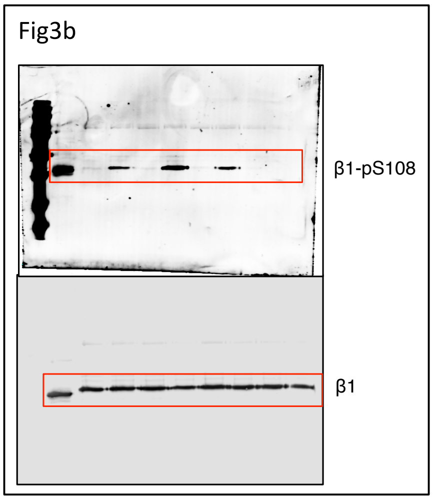
Fig3b
β1-pS108
β1

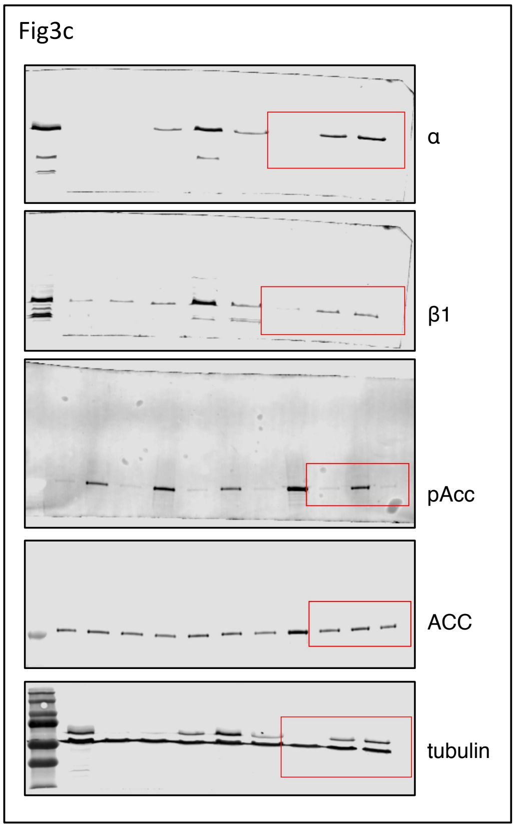
Fig3c
α
β1
pAcc
ACC
tubulin

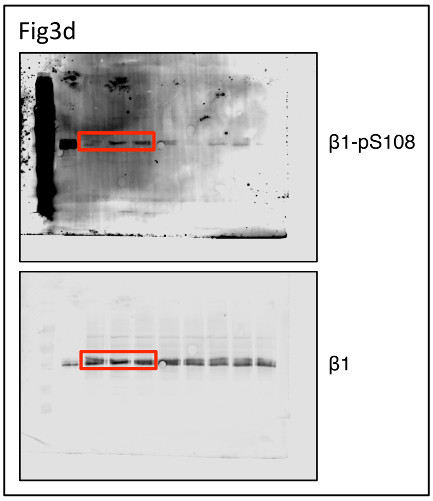
Fig3d
β1-pS108
β1

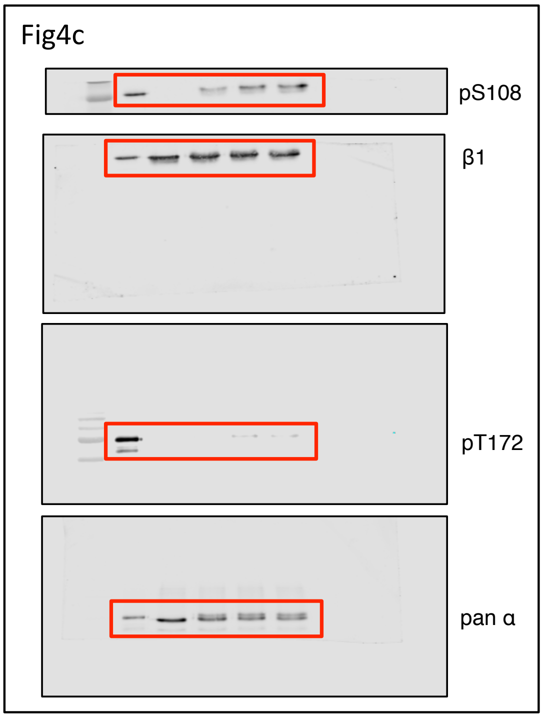
Fig4c
pS108
β1
pT172
pan α

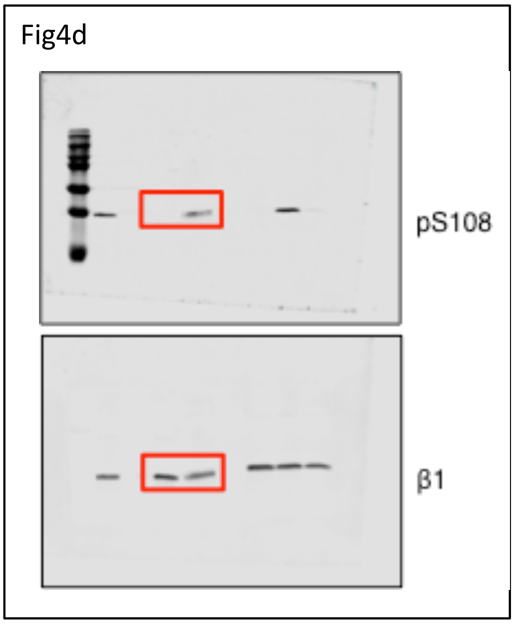
Fig4d
pS108
β1

Fig5a

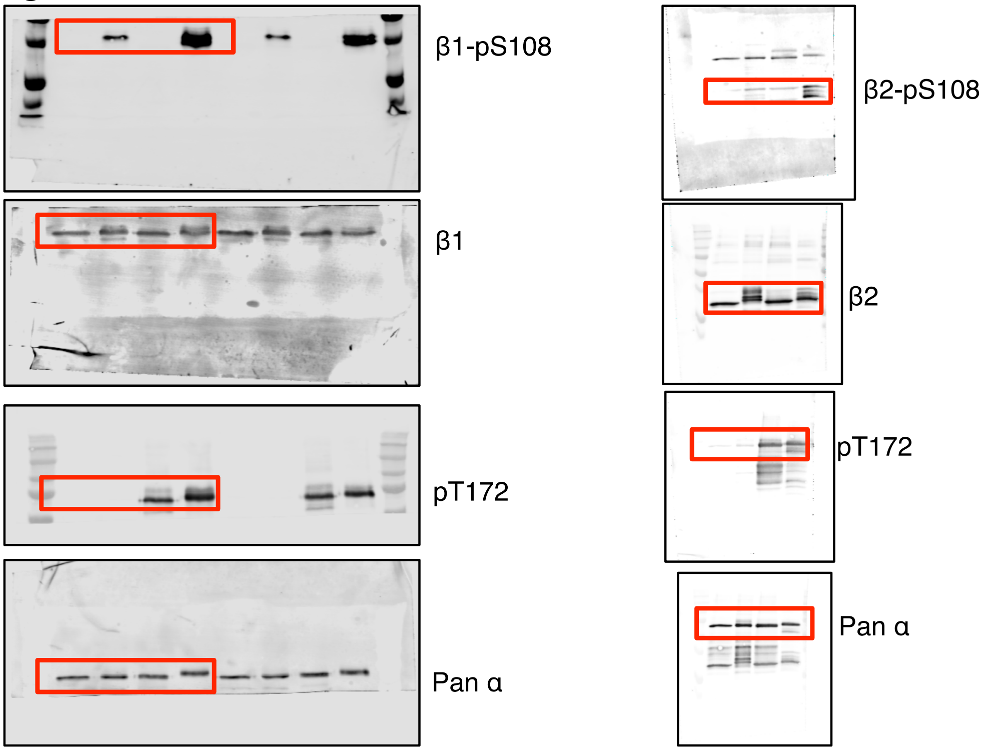
β1-pS108
β1
pT172
Pan α
β2-pS108
β2
pT172
Pan α

Fig5b

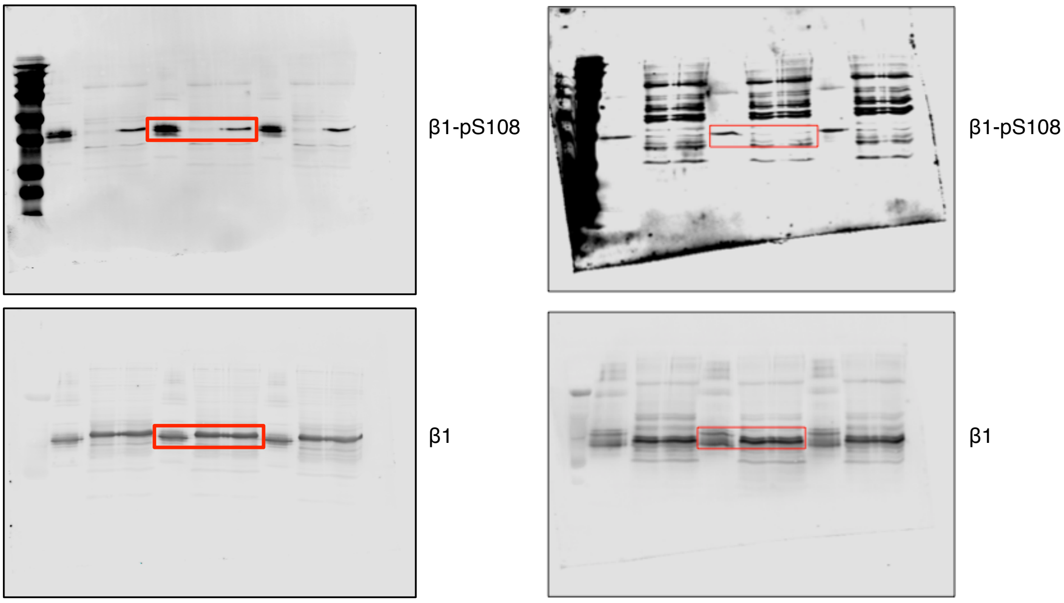
β1-pS108
β1
β1-pS108
β1

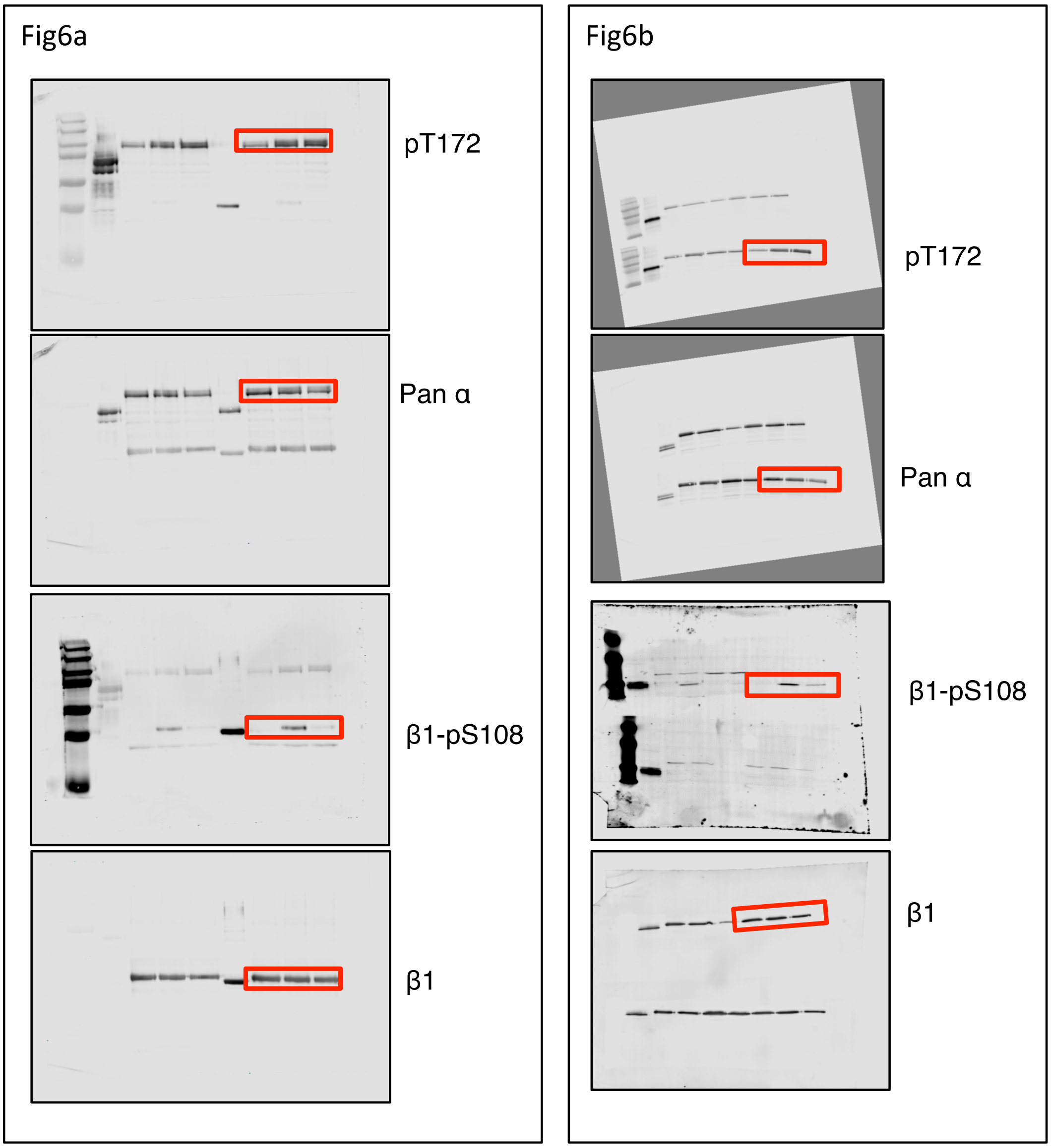
Fig6a
pT172
Pan α
β1-pS108
β1
Fig6b
pT172
Pan α
β1-pS108
β1

Fig6c

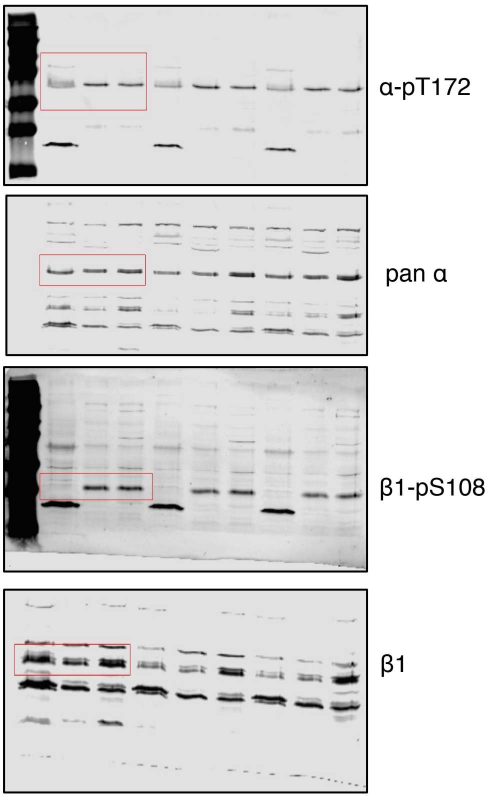

Fig6d

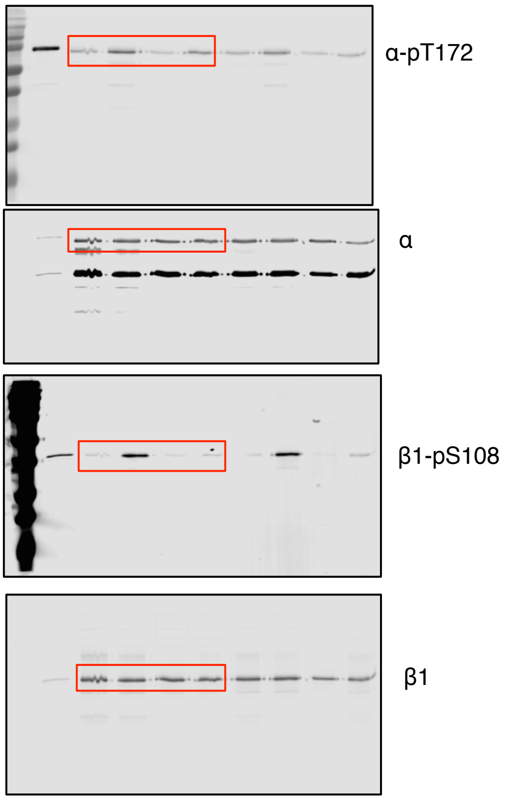

Fig7b

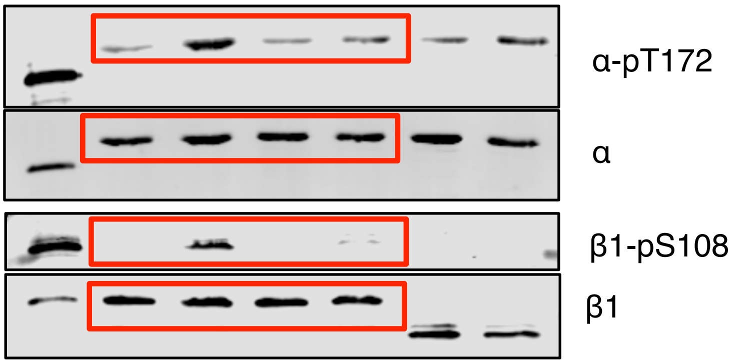

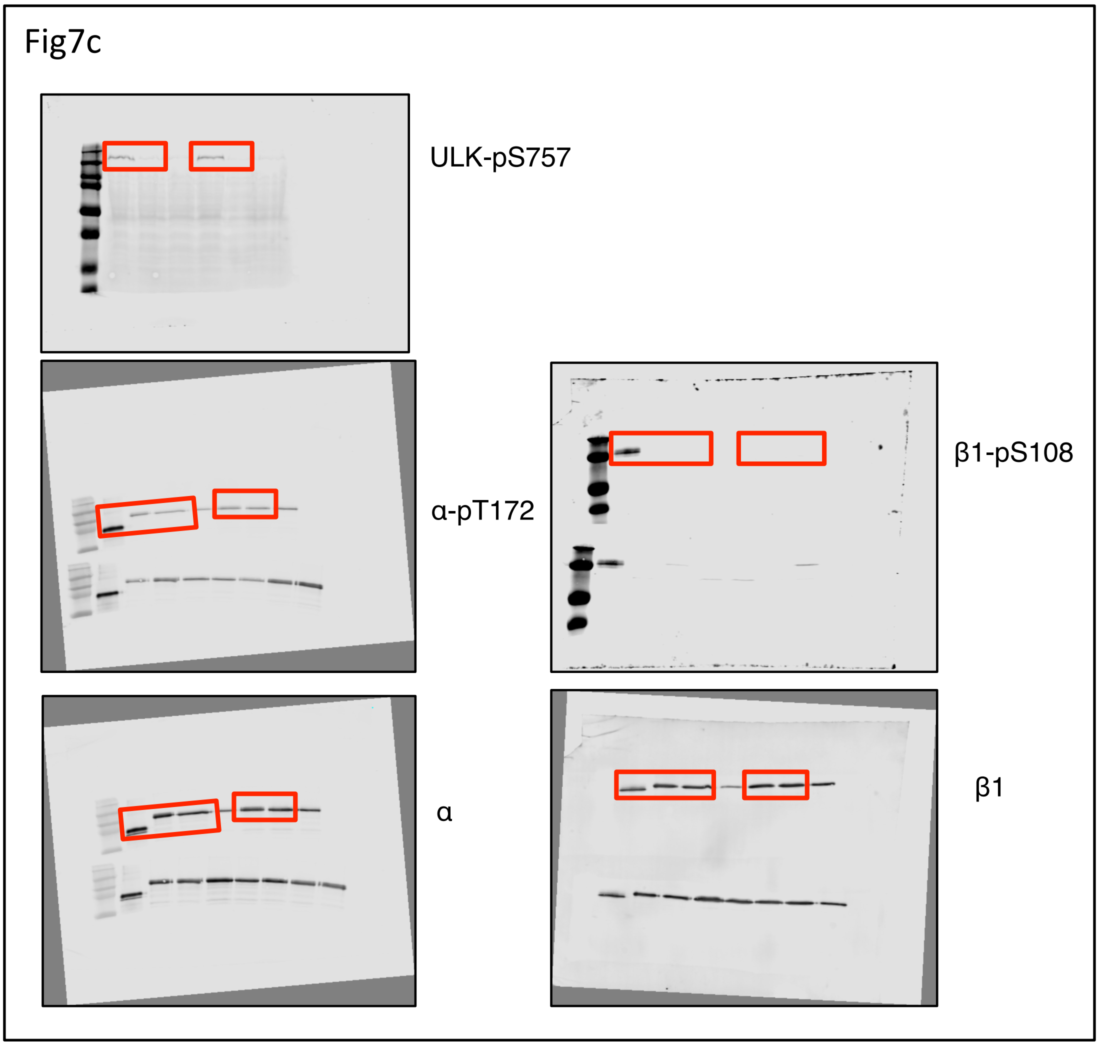
Fig7c
ULK-pS757
α-pT172
β1-pS108
α
β1

Fig7d

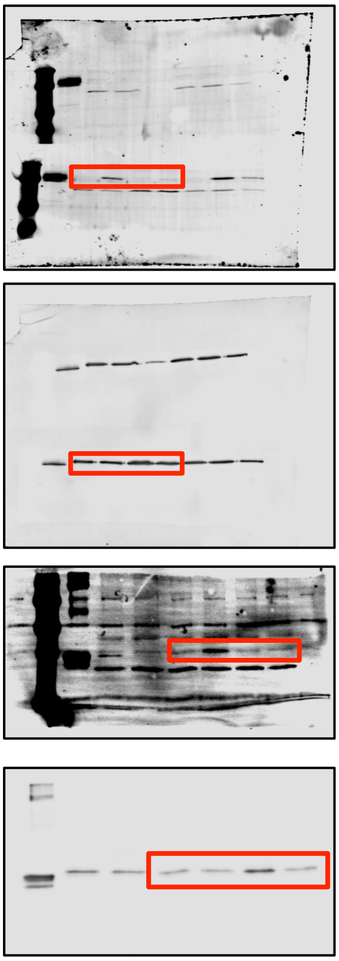

Fig7e

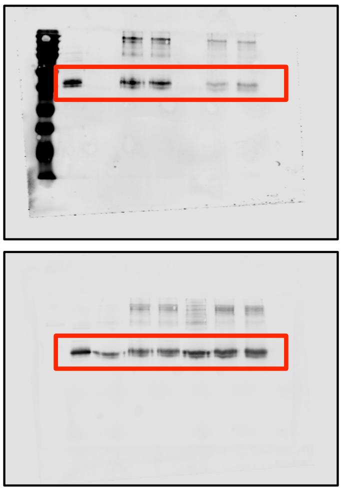

Fig8a

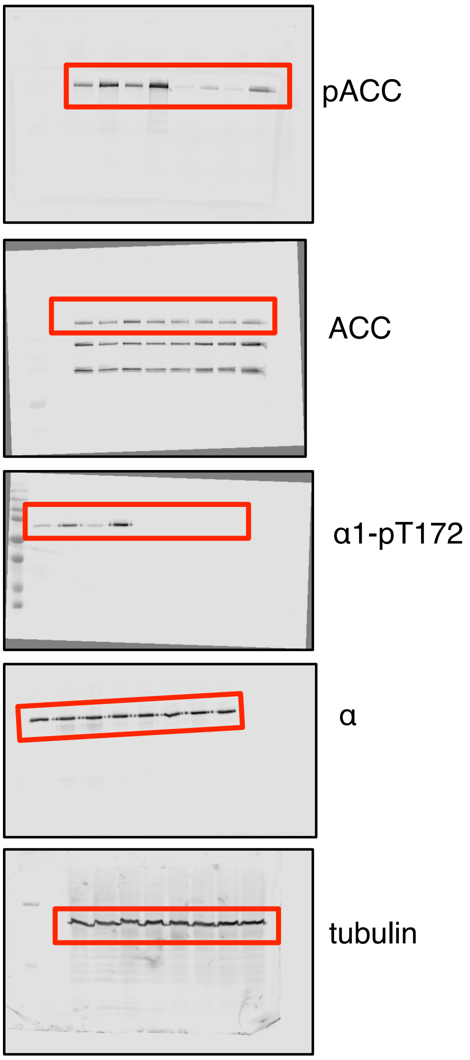

Fig8b

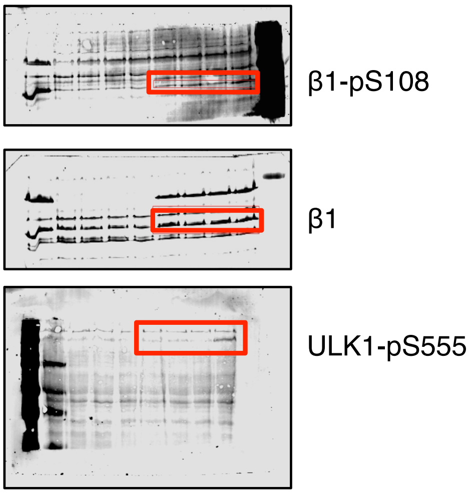

Supplementary Fig1b

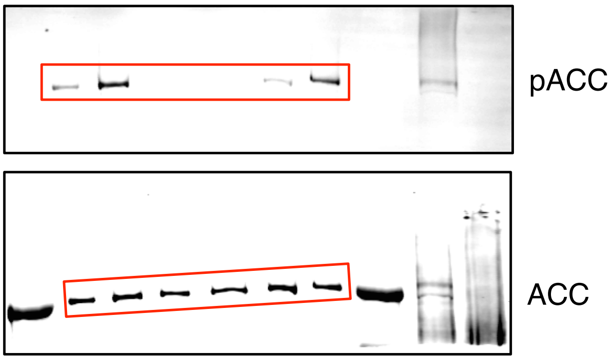

Supplementary Fig1c

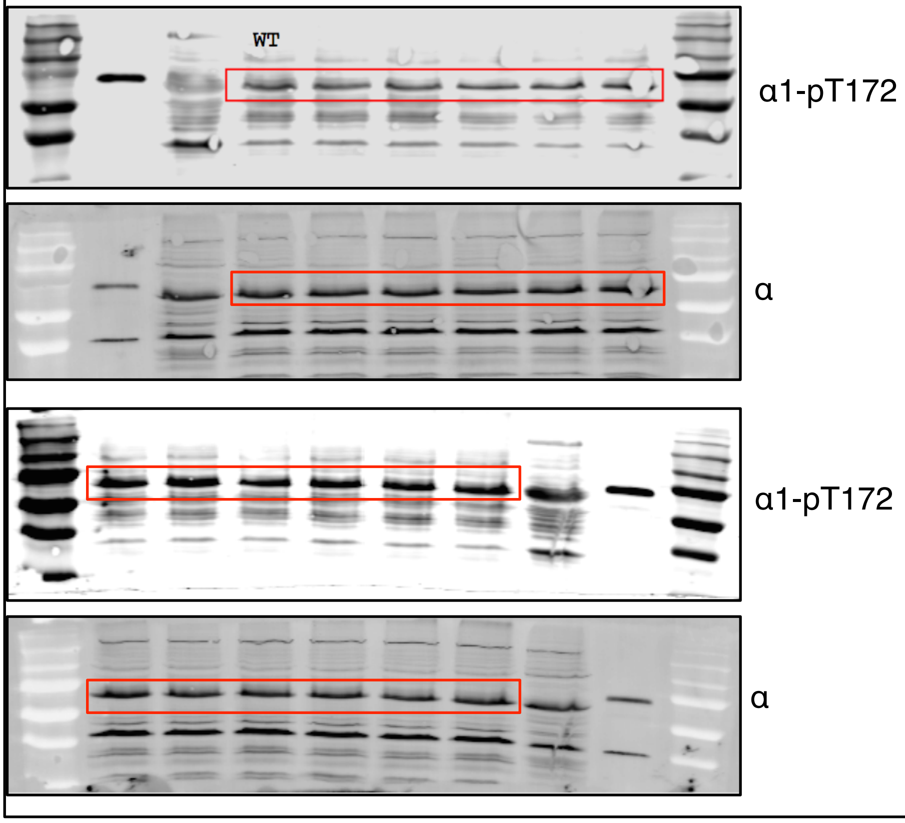

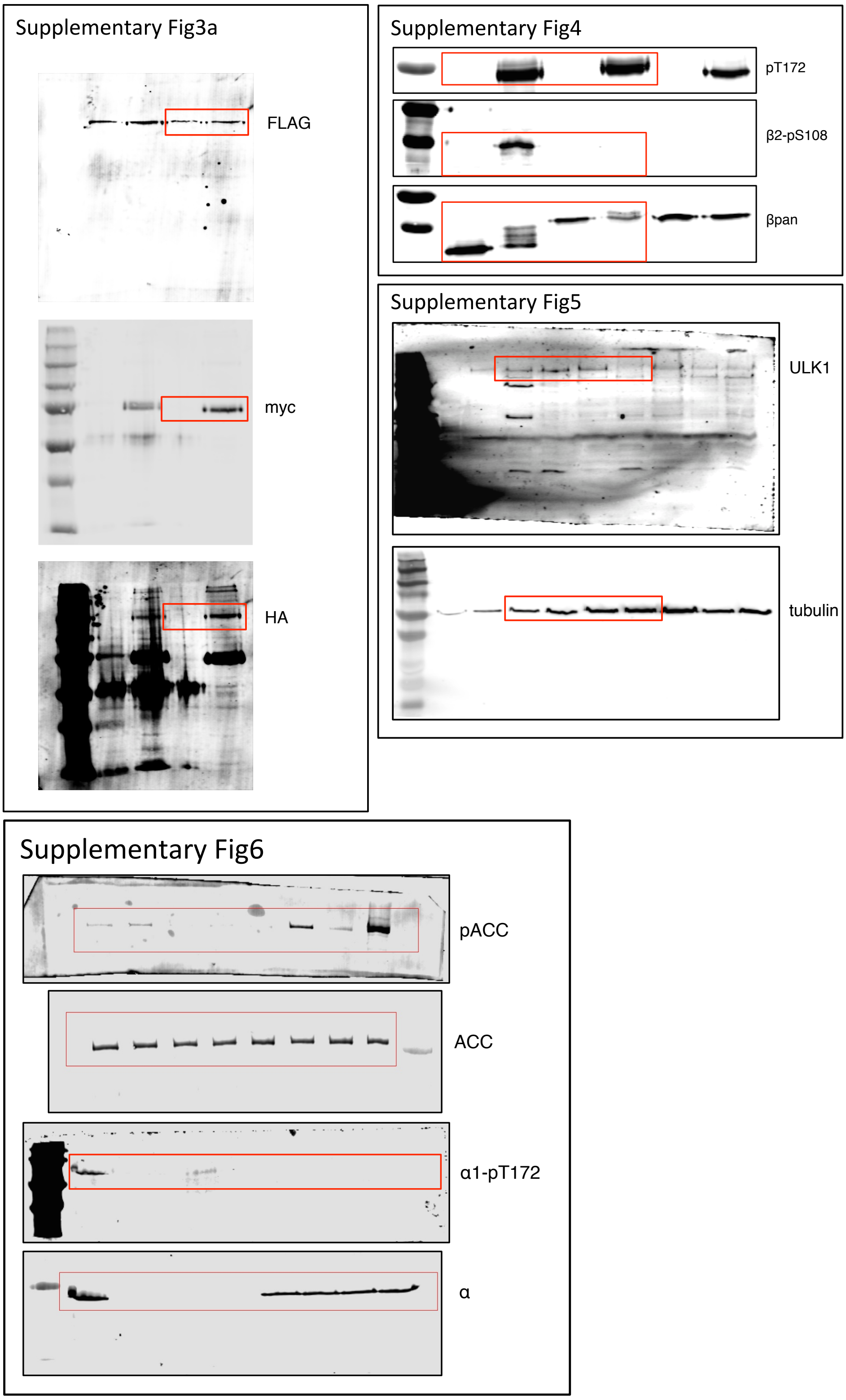

Supplementary Figure 7. Uncropped blots corresponding to main and supplementary figures as indicated.

identifier (mouse)	protein	phosphosite
OSB11_S179	Oxysterol-binding protein-related protein 11	KSRSFsLASS
PAK2_S141	p21-activated protein kinase	KQKYLsFTPP
_S152		KDGFPsGTPA
DOCK7_S452	Dedicator of cytokinesis protein 7	LERTTsGDDA
ANS1A_S663	Ankyrin repeat/SAM domain-containing protein 1A	GSRSEsLSNC
PALM_T141	Paralemmin-1	VNAQQtPLGT
_T145		QTPLGtPKEN
TB182_S1133	182 kDa tankyrase-1-binding protein	AGLSPsRKSG
TR150_S238	Thyroid hormone receptor-associated protein 3	SASRAsVSDL
_S243		SVSDLsPRER
TCOF_S593	Treacle protein	SASLSsPALA
_S1191		QKRKLsGDLE
GLCE_S73	D-glucuronyl C5-epimerase	IAKQQsEEAF
IBTK_S1046	Inhibitor of Bruton tyrosine kinase	PRDLQsPDFT
ML12B_T19	Myosin regulatory light chain 12B	RPQRAtSNVF
_S20		PQRATsNVFA
CND1_S1320	Condensin complex subunit 1	LQPLTsVDSD
_S1323		LTSVDsDNDF
BIN1_S296	Myc box-dependent-interacting protein 1	EKGNKsPSPP
NHRF1_S285	Na(+)/H(+) exchange regulatory cofactor NHE-RF1	LARSAsSDTS

Supplementary Table 1. Details of significantly altered phosphosites in β1-S108E expressing cells. Underlined residues indicate concensus with the AMPK substrate recognition motif (P-5: Ile, Leu, Met; P-4/P-3/P-2: Arg; P+4: Ile, Phe, Leu (www.kinasenet.ca)).

No.	Protein Kinase	Activity (cpm) Corrected
1	AMPK (A1/B2/G1)	478,776
2	AMPK (A2/B1/G2)	459,050
3	AMPK (A1/B2/G2)	451,721
4	AMPK (A1/B1/G2)	438,106
5	AMPK (A1/B1/G1)	385,831
6	AMPK (A2/B1/G1)	356,911
7	AMPK (A2/B2/G1)	344,901
8	AMPK (A2/B1/G3)	327,772
9	AMPK (A2/B2/G2)	316,245
10	AMPK (A1/B1/G3)	312,857
11	AMPK (A1/B2/G3)	310,149
12	BRSK1	302,949
13	BRSK2	277,167
14	NEK2	269,802
15	ULK1	213,669
16	ULK2	205,507
17	MLK1	202,048
18	PKC alpha	197,873
19	TSSK2	186,641
20	TAOK1	182,953
21	IRAK4	148,291
22	NEK9	141,782
23	TTBK1	118,176
24	TTBK2	92,191
25	TSSK1B	89,196
26	ROCK2	85,906
27	NEK7	77,797
28	NEK6	72,437
29	TAOK3	70,847
30	GCK	60,622
31	ZAK	56,640
32	EIF2AK3	54,243
33	MARK3	52,548
34	RIPK3	50,491

Supplementary Table 2. Top kinase candidate hits using S108tide synthetic peptide substrate. Kinase screens (IKPT service) performed by Kinexus, Canada. Most assays were performed for 15 min duration, 50 μM [γ-^{33}P]ATP, in a 25 μl reaction volume at 30°C. Activity corrected to background, >50,000 cpm threshold.

vector	protein	sequence
pcDNA3	AMPKα1-flag	For: 5' ggtactcgagatggcgacagccgagaagcag 3'
		Rev: 5’ ggtagaattctcacttatcgtcatcgtctttgtaatcgcctccttgtgcaagaattttaattag 3'
	AMPKα2-flag	For: 5' ggtactcgagatggctgagaagcagaagc 3'
		Rev: 5' gcataagctttcacttatcgtcatcgtctttgtaatcgcctccacgggctaaagtag 3'
LeGO-iG2	AMPKα1-flag	For: 5' ggtagaattcatggcgacagccgagaagcag 3'
		Rev: 5’ ggtagcggccgctcacttatcgtcatcgtctttgtaatcgcctccttgtgcaagaattttaattag 3'
	AMPKα2-flag	For: 5' ggtagaattcatggctgagaagcagaagcacgacg 3'
		Rev: 5' ggtagcggccgctcacttatcgtcatcgtctttgtaatcgcctccacgggctaaagtag 3'
	AMPKβ1-flag	For: 5' ggtaggatccatgggcaataccagcagtgagc 3'
		Rev: 5' cgtcgcggccgctcacttatcgtcatcgtctttgtaatcgcctcctatgggcttgtataacaagg 3'

mutation	sequence
AMPKβ1-S108A	For: 5' cccctcaccagagcccacaataactttgtagc 3’
AMPKβ1-S108E	For: 5' cccctcaccagagaacacaataactttgtagc 3'
AMPKα1-D141A	For: 5' ggtggtccatagagctttgaaacctgaaaatg 3'
AMPKα2-T172A	For: 5' ggtgaatttctgagagctagttgcggatctcc 3'
AMPKα1-C176S	For: 5' gaatttttaagaacaagtagtggctcacccaactatgc 3’

Supplementary Table 3. Details of primers used in this study. Oligonucleotides are displayed from 5’ to 3’. Where primers were used for PCR amplification, both forward (Fwd) and reverse (Rev) sequences are shown. Where primers were used in mutagenesis reactions, only the sense strand sequence is shown, and nucleotide changes are underlined.
